# *Arabidopsis*: An Adequate Model for Dicot Root Systems?

**DOI:** 10.3389/fpls.2016.00058

**Published:** 2016-02-05

**Authors:** Richard W. Zobel

**Affiliations:** Plant Science Research Unit, Agricultural Research Service, United States Department of Agriculture, RaleighNC, USA

**Keywords:** *Arabidopsis*, roots, root classes, model plants, root system architecture (RSA)

## Abstract

The *Arabidopsis* root system is frequently considered to have only three classes of root: primary, lateral, and adventitious. Research with other plant species has suggested up to eight different developmental/functional classes of root for a given plant root system. If *Arabidopsis* has only three classes of root, it may not be an adequate model for eudicot plant root systems. Recent research, however, can be interpreted to suggest that pre-flowering *Arabidopsis* does have at least five (5) of these classes of root. This then suggests that *Arabidopsis* root research can be considered an adequate model for dicot plant root systems.

## Introduction

Root system architecture (RSA) may be a key to the further improvement of crop productivity (Lynch, 2007). A significant amount of research is being carried out on *Arabidopsis* roots as a model system (Benfy et al., 2010). This research includes both morphological, anatomical, and physiological/molecular studies. Historically this research has referred to two types of *Arabidopsis* root, the primary root and its lateral roots. More recent studies of adventitious roots on *Arabidopsis* have begun to elucidate a more complex rooting system. Zobel (2011) has suggested that both monocot and eudicot root systems are made up of 8 or more genetically and functionally distinct classes of root. Does the *Arabidopsis* root system architecture fit this model or is it a subset with only 2–3 classes of root?

First, what is Root System Architecture? Two systems for describing RSA have been put forward. Fitter (1987) described a method that classified the architecture of a root system, or parts of a root system, as a “topology.” The topological patterns he demonstrated were shown to coincide well with concepts of efficient root exploration for nutrients (Fitter and Stickland, 1991; Fitter et al., 1991). This topology normally describes roots of very young plants or a single major root with it’s attendant laterals. Topology, as defined in dictionaries, however, is the study of the topography of an object. Zobel (2011) describes the topography of an intact whole plant root system, in terms of the major structures that are included with in that topography. Zobel’s RSA, relative to a given plant, can be further defined (refined) through studies of root angle, root diameter, growth rate, differential function, etc. Fitter’s topology describes visual patterns of lateral root development within that topography and thus is also a refining element that can be explored independent of the overall topography.

Second, what is the significance of Zobel’s (2011) RSA (**Table 1**). The classification proposed was based on documented differential genetic control of the initiation and development of different roots with in the root system of both monocot and eudicot plants. Combined with this was evidence that some of these classes of root are functionally distinct. For instance, for both soybean (*Glycine max* L.) and maize (*Zea mays* L.) the tap root and basal roots can have different abilities to penetrate compacted soil or aluminum toxic soil (Zobel, 2011). These abilities vary with cultivar, such that if a plant breeder desires cultivar of soybean that can penetrate Aluminum toxic soil, that breeder would need to identify a cultivar which has the ability for the tap root to penetrate the toxic soil, and another cultivar that has the ability for the basal roots to penetrate the toxic soil. The breeder would then need to hybridize the two cultivars and select from among the offspring a line which has the ability for both the tap root and basal roots to penetrate the toxic soil. The same requirements hold for the other classes of root. This necessitates that function (and molecular patterns) of different classes of root be studied in isolation of the other classes of root. If *Arabidopsis* is to be a useful model plant, these different classes of root need to be identified within its RSA so they can be studied with the powerful tools already developed for this species.

**Table 1:** Table of root nomenclatures, modified from Zobel (2011).

Major framework classes	Sub class	Abbreviation	Class #
Tap root (first root to emerge)		TRT	1
Basal root (develops from the hypocotyl)	Basal root (just above the base of the taproot) often seminal in origin	BRT	2
	Hypocotyl root (on the middle to upper hypocotyl)	HRT	3
Shoot-Borne root (develops from shoot tissues)	Coleoptile (cotyledon) node roots	CNRT	4
	Roots on nodes above the coleoptile node or cotyledons	SNRT	5
	Roots on internodes above the coleoptile node or cotyledons	SIRT	6
Lateral root (branches from another root)	Branch roots on the tap root, basal, hypocotyl or other lateral roots	Prefix main root abbreviation with an L: i.e., LTRT or LHRT (a lateral branching from another lateral = LTRT2 or LTRT3 dependent on branching level, counting from main axis)	7
	Branch roots on shoot-borne roots	Begins with L: i.e., LSNRT (similar to above)	8

*Items in italics (classes 4, 5, 8) have not been demonstrated on *Arabidopsis* at this time. Class 4 has yet to be demonstrated in eudicots.*

## Discussion

Although *Arabidopsis* is generally considered “the model plant” for eudicots, it’s root system topography (RSA) has been generally accepted to have only two classes of root: tap root (primary root), and lateral roots. This conclusion was probably based on the early research with *Arabidopsis* roots being restricted to seedling plants, and the classical definition of root system architecture (Esau, 1965). Falasca and Altamura (2003) demonstrated that a third possible class of root, “adventitious,” was distinct from lateral roots, thus raising the number of root classes for *Arabidopsis* to three. Unfortunately such a conclusion, when presented with Zobel’s (2011) conclusion, suggests that *Arabidopsis* may not be a fully representative model for eudicot root systems.

More recent research with “adventitious” roots of somewhat older *Arabidopsis* plants provides some clarity to this situation. The term “adventitious” has two different competing definitions, the strict definition and the common usage definition. The strict definition is that adventitious roots do not arise from pericycle cells. The more general definition is: any root that is not a lateral branch of another root. Because neither of these definitions allows the reader to easily ascertain the actual site of origin of the roots in question, in 1996 the International Society for Root Research (ISRR), declared the term “adventitious” obsolete, and replaced it with term “shoot-borne” (Zobel and Waisel, 2010). Falasca and Altamura (2003) demonstrated that roots arising from the hypocotyl (i.e., non-root tissue) were derived from a pericycle in a manner identical with that of lateral roots of the tap root. Therefore, hypocotyl-borne roots of *Arabidopsis*, are not adventitious (*sensu stricto*). Furthermore, Verstraeten et al. (2014) have demonstrated that these hypocotyl-borne roots are distinct from lateral roots in terms of their molecular based initiation patterns. Similar roots from the hypocotyl of beans (*Phaseolus vulgaris* L.) have been shown to be important for adaptation to low phosphorus conditions (Lynch and Brown, 2001). Welander et al. (2014) took the study of adventitious roots in *Arabidopsis* one step further and demonstrated molecular differentiation between hypocotyl-borne roots and shoot-borne roots. To add to this, a visual assessment of Celenza et al. (1995 - Figures 9A,C), suggests that the *Arabidopsis* mutant *alf1-1* is a proliferate hypocotyl-borne root mutant. Thus it would appear that *Arabidopsis* has both hypocotyl roots (HRT) and shoot-borne roots (SIRT) from Zobel’s (2011) topography (**Table 1**).

Images of wild type *Arabidopsis*, often show one or more large roots originating at the hypocotyl/taproot junction (i.e., basal; base of the root and base of the shoot; cf Figures 1D,E of King et al., 1995; Figure 3B “WT” in Pacurar et al., 2014; and Figure 1 in Verstraeten et al., 2014; for example). Li and Gao (2011) discuss stimulation of root initiation at the junction of the tap root with the hypocotyl of *Arabidopsis* by a coumarin derivative (4-nethyllumbelliferone). Combining this with the visual observations, it can be suggested that *Arabidopsis* has a distinct class of roots originating at the base of the shoot/root. Both the ISRR (Zobel and Waisel, 2010) root classification system and Zobel’s (2011) topography, classify roots from this position as basal roots (BRT). It has been demonstrated in bean that the angle of BRT outgrowth from the axis of the shoot can be critical for efficient phosphorus uptake (Lynch and Brown, 2001).

The topography of the *Arabidopsis* root system appears to fit with the typical topography of eudicot root systems as presented by Zobel (2011). The root classes of *Arabidopsis*, using the terms of Zobel (2011, Table 1), thus are: tap root (TRT), basal root (BRT), hypocotyl root (HRT), internodal shoot-borne root (SIRT), and lateral root (LRT). This commonality with other eudicots, strengthens the argument for *Arabidopsis* root systems being model dicot root systems. Continued molecular and developmental research with these different root classes should help to solidify or adjust the proposed classification of Zobel (2011) for the functional topography of eudicot root systems.

## Author Contributions

The author confirms being the sole contributor of this work and approved it for publication.

## Conflict of Interest Statement

The author declares that the research was conducted in the absence of any commercial or financial relationships that could be construed as a potential conflict of interest.
